# Identification of Trenbolone Metabolites Using Hydrogen Isotope Ratio Mass Spectrometry and Liquid Chromatography/High Accuracy/High Resolution Mass Spectrometry for Doping Control Analysis

**DOI:** 10.3389/fchem.2020.00435

**Published:** 2020-05-20

**Authors:** Marlen Putz, Thomas Piper, Mario Thevis

**Affiliations:** Center for Preventive Doping Research, Institute of Biochemistry, German Sport University Cologne, Cologne, Germany

**Keywords:** gas chromatography thermal conversion isotope ratio mass spectrometry (GC-TC-IRMS), liquid chromatography high resolution mass spectrometry (LC-HRMS), human metabolism, steroids, phase-II conjugates, pseudo MS^3^ product ion mass spectra, sports drug testing, *in vivo* metabolism

## Abstract

Trenbolone is a synthetic anabolic-androgenic steroid, which has been misused for performance enhancement in sports. The detection of trenbolone doping in routine sports drug testing programs is complex as methods utilizing gas chromatography/mass spectrometry are complicated by unspecific derivatization products and artifacts, and liquid chromatography/mass spectrometry-based assays have shown to allow for comparably high limits-of-detection only. The number of previously reported metabolites in human urine is limited, and most analytical methods rely on targeting epitrenbolone, trenbolone glucuronide, and epitrenbolone glucuronide. In order to probe for the presence of additional trenbolone metabolites and to re-investigate the metabolism, an elimination study was conducted. One single dose of 10 mg of 5-fold deuterated trenbolone was administered to a healthy male volunteer and urine samples were collected for 30 days. For sample processing, published protocols were combined considering unconjugated, glucuronic acid-, sulfo- and alkaline-labile conjugated steroid metabolites. The sample preparation strategy consisted of solid-phase extractions, liquid-liquid extractions, metabolite de-conjugation, HPLC fractionation, and derivatization. Analytical methods included gas chromatography/thermal conversion/hydrogen isotope ratio mass spectrometry combined with single quadrupole mass spectrometry as well as liquid chromatography/high accuracy/high resolution mass spectrometry of the hydrolyzed and non-hydrolyzed samples. Twenty deuterium-labeled metabolites were identified including glucuronic acid-, sulfo- and potential cysteine-conjugates, and characterized by parallel reaction monitoring experiments yielding corresponding product ion mass spectra. Main metabolites were attributed to trenbolone-diol and potential trenbolone-diketone derivatives excreted as glucuronic acid and sulfo-conjugated analytes with detection windows of 5, respectively 6 days. Further characterization was conducted with pseudo MS^3^ experiments of the intact conjugates and by comparison of resulting product ion mass spectra with reference material.

## Introduction

**Figure 1:**
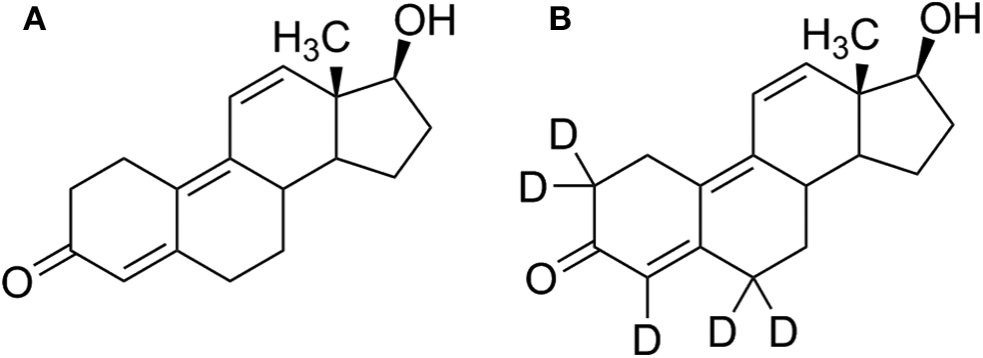
Structure formulae of **(A)** trenbolone and **(B)** d_5_-trenbolone used for the excretion study.

Trenbolone (Tren) belongs to the class of synthetic anabolic-androgenic steroids (AAS) and is structurally characterized by a 4,9,11-triene-3-one structure composing a highly conjugated π-electron system (Figure 1). The significant anabolic properties of Tren resulting in increased muscle size and strength have generated an incentive for illicit applications including doping and livestock breeding. In sports, the use of trenbolone has been prohibited by the World Anti-Doping Agency (WADA) at all times, categorized under S1 1. (anabolic androgenic steroids) in the Prohibited List (WADA, 2020). According to WADA's annual statistics, anabolic agents are the most frequently misused substance group in sports with a total of 1,823 adverse analytical findings (AAFs) in 2018. Within this group, Tren occurrences account for 6% (WADA, 2019). The statistics however can only reflect cases of detectable Tren and does not conclusively address the question whether Tren is less favored by users of AAS or if available detection strategies do not offer the required analytical retrospectivity.

For that reason the objective of this project was to re-investigate the metabolism of Tren in order to probe for metabolic products potentially supporting the extension of the detection window. The number of previously reported Tren metabolites is scarce, and for doping control purpose the analysis focuses at present on the main human urinary metabolites epitrenbolone (EpiTREN), epitrenbolone glucuronide (EpiTREN Glu), and trenbolone glucuronide (TREN Glu) (De Boer et al., 1991; Schänzer, 1996). Regarding the detection windows, two data sets have been published spanning from approximately 3 days (Spranger and Metzler, 1991) to 32 days (Sobolevsky and Rodchenkov, 2015). Besides glucuronides, sulfates (Rzeppa et al., 2015), and cysteine conjugates (Sobolevsky and Rodchenkov, 2015) of Tren and Epitren were reported. Cysteine conjugates are produced by phase-II metabolism, where the tripeptide glutathione is covalently bound via the sulfur atom by glutathione transferase and, subsequently, glutamate and glycine are eliminated. In general, cysteine conjugates are hydrolyzed employing alkaline conditions (Blair, 2006; Fabregat et al., 2010, 2011, 2013; Pozo et al., 2010), but the cysteine conjugate of trenbolone described by Sobolevsky and Rodchenkov (2015). was found to be stable during alkaline hydrolysis and was analyzed by high performance liquid chromatography electrospray ionization tandem mass spectrometry (HPLC-ESI-MS/MS) as in-source fragment in ESI negative mode and as intact conjugate in ESI positive mode (Sobolevsky and Rodchenkov, 2015). During *in-vitro* studies, several monohydroxylated metabolites, and trenbolone-diketone were generated (Metzler and Pfeiffer, 2001; Kuuranne et al., 2008).

Nowadays, LC-MS-based methods are commonly used for the analysis of Tren and its metabolites (Thevis et al., 2005a, 2009; Tudela et al., 2015) as GC-MS-based methods were found to be of limited utility due to derivatization artifacts and low thermal stability of the target analytes (De Boer et al., 1991; Ayotte et al., 1996; Casademont et al., 1996; Marques et al., 2007; Brun et al., 2011).

For systematic metabolism studies, a method for metabolite identification using hydrogen isotope ratio mass spectrometry was developed and successfully applied for the first time in 2013 (Thevis et al., 2013). The fundamental principle is analogous to metabolism studies using radioactively labeled compounds (Sano et al., 1976). The compounds can be detected selectively because of their isotopic labeling by measuring the radioactivity in case of tritium or ^14^C labeled compounds or by measuring the hydrogen isotope ratios in case of deuterium labeled compounds.

Hydrogen isotope ratios are determined by gas chromatography/thermal conversion/isotope ratio mass spectrometry (GC-TC-IRMS). The organic compounds are converted under reducing conditions to CO and N_2_ as well as molecular hydrogen (H_2_), respectively the deuterated isotopologe HD. After ionization, detection is accomplished using *m*/*z* 2 for ${\text{H}}_{2}^{+}$ and *m*/*z* 3 for HD^+^ by Faraday cups with different amplification factors (factor 1000 difference). Since the natural hydrogen abundance amounts on average to 99.985% for H and 0.015% for D (Dunn and Carter, 2018), comparable signals for ${\text{H}}_{2}^{+}$ and HD^+^ are obtained for samples at natural abundance, while deuterated compounds lead to a significant increase of signals at *m*/*z* 3. Compounds resulting in diagnostic HD^+^ signals are subsequently comprehensively characterized by gas chromatography/electron ionization/high accuracy/high resolution mass spectrometry (GC-EI-HRMS). This concept has been proven in several studies (Thevis et al., 2013; Piper et al., 2016a,b, 2018, 2019).

Within this project, liquid chromatography/electrospray ionization/high accuracy/high resolution mass spectrometry (LC-ESI-HRMS) was applied for further characterization of trenbolone and its metabolites after GC-TC-IRMS analysis. Twenty metabolites were identified with a detectability of up to 6 days. Four metabolites exhibiting the longest detection windows were characterized by parallel reaction monitoring (PRM) experiments and comparison to reference material.

## Materials and Methods

### Chemicals and Steroids

Trenbolone reference material and the internal standard 2,2,4,6,6-d_5_-trenbolone were purchased from Toronto Research chemicals (Toronto, Canada), and epitrenbolone from the National Measurement Institute (Sydney, Australia). Steroid reference material for HPLC separation including ETIO (etiocholanolone), A (androsterone), T (testosterone), and PD (pregnanediol) was supplied by Sigma-Aldrich, and 11 K (11-ketoetiocholanolone), 5a (5α-androstanediol), and 5b (5β-androstanediol) were obtained from Steraloids (Newport, RI). Chromabond C18 solid-phase extraction (SPE) cartridges (500 mg, 6 mL) were purchased from Macherey & Nagel (Düren, Germany) and β-glucuronidase from Escherichia coli (140 U/mL) from Roche Diagnostics (Mannheim, Germany). Ultrapure water was prepared by a Barnstead™ GenPure™ xCAD Plus system (Thermo, Germany). All reagents and solvents were of analytical grade. Acetonitrile (ACN), formic acid (FA), methanol (MeOH), tert-butyl methyl ether (TBME), cyclohexane, pyridine, sodium hydroxide (NaOH), sulfuric acid (H_2_SO_4_), glacial acetic acid, and potassium tri-sec-butylborohydride (1 M in THF) were provided by Merck (Darmstadt, Germany). Acetic anhydride was supplied by Sigma Aldrich (Taufkirchen, Germany) and tetrahydrofuran (THF) by VWR (Darmstadt, Germany). *N*-methyl-*N*-trimethylsilyltrifluoroacetamide (MSTFA) was purchased from Chemische Fabrik Karl Bucher (Waldstetten, Germany).

### Excretion Study

An excretion study was conducted in order to re-investigate the trenbolone metabolism. Following written informed consent, 10 mg of 5-fold deuterated trenbolone (Figure 1) dissolved in ultra-pure water/EtOH (80:20, *v*/*v*) was orally administered to one healthy male volunteer (43 years, 84 kg) who declared not to have used any medication or nutritional supplements during this study and for a wash-out period of at minimum 3 month (any compounds), respectively 6 month (deuterated compounds) before the study. Three blank samples were collected pre-administration, and post administration samples were collected for up to 30 days. During the first 48 h after trenbolone intake, every urine was collected. From day three until day five, two to three urine samples per day were collected, and afterwards only the first morning urine was sampled until the end of the study. The administration study was approved by the Ethics Committee of the National Institute of Sports of Romania (Bucharest, Romania, #2283, 2016).

### Analysis of Hydrolyzed Steroids

#### Sample Preparation

**Figure 2:**
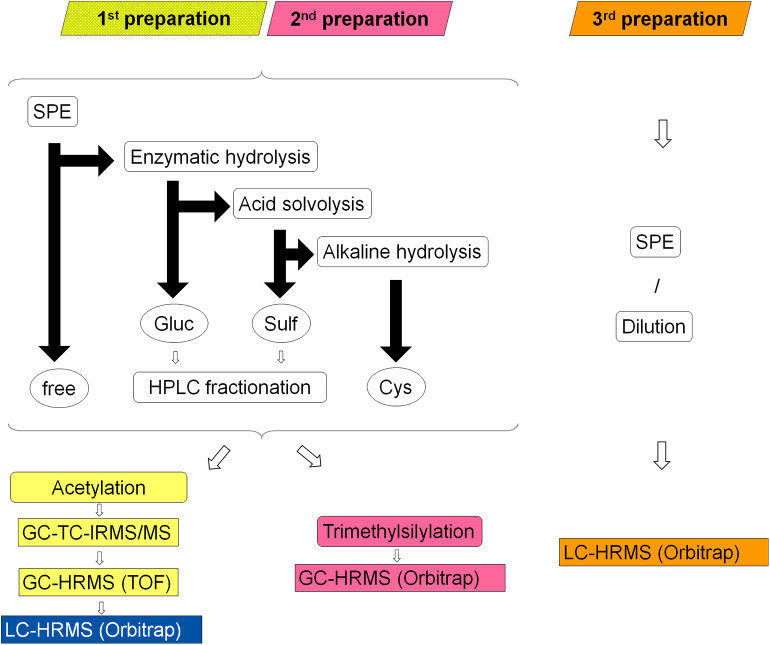
Schematic overview of the three different sample preparations colored in yellow/blue (1^st^), pink (2^nd^) and orange (3^rd^); rounded corner boxes indicate sample processing steps, circles indicate former conjugation forms of the liberated steroids (free = unconjugated steroids, Gluc = glucuronides, Sulf = sulfates, Cys = cysteine conjugates), black arrows represent the sample separation by LLE: horizontal arrow represent processing of the aqueous layer and vertical arrows of the organic layer, rectangular boxes indicate the applied analytical systems.

An extensive sample preparation was required in order to reach adequate purity and undecomposed volatility of the metabolites for GC-TC-IMRS analysis. Urine samples were prepared according to established protocols for isotope ratio analysis of steroids. Here, every sample is separated into four main fractions: unconjugated steroids, glucuronic acid conjugates (Piper et al., 2008), sulfo-conjugates (Piper et al., 2010), and cysteine conjugates (Fabregat et al., 2010, 2011, 2013; Pozo et al., 2010). Subsequently, the fractions of glucuronides and sulfates are further divided by HPLC into seven sub-fractions (Thevis et al., 2013). Sample preparation and analysis is summarized in Figure 2.

For each sample, a volume of 20 mL urine was required. First, two C-18 SPE cartridges per sample were pre-conditioned with 2 mL of MeOH and subsequently washed with 2 mL of water. Then, every sample was split and 10 mL of urine were applied to each cartridge, which was subsequently washed with 2 mL of water, and finally eluted with 2 mL of MeOH. Both eluates of each sample were combined and evaporated to dryness under a gentle stream of compressed air at 50°C. Following evaporation, samples were reconstituted with 1 mL of an aqueous 0.2 M sodium phosphate buffer at pH 7, and a liquid-liquid-extraction (LLE) step with 5 mL of TBME was performed. Therefore, the mixture was shaken for 5 min and subsequently centrifuged for 5 min at 600 g before separating both layers. The organic layer yielded the fraction of the unconjugated steroids (fraction f; free).

The remaining aqueous layer was incubated with 100 μL of β-glucuronidase at 50°C for 1 h. To terminate hydrolysis, 500 μL of an aqueous 20% potassium carbonate buffer (pH 10) were added. In order to extract the liberated steroids, a second LLE step with TBME was performed and the resulting organic layer contained the former glucuronic acid conjugates (fraction Gluc). Then, the pH of the aqueous layer was adjusted to 5 with glacial acetic acid, and purified by SPE as described above. After evaporation, samples were incubated with 2.5 mL of EtOAc/MeOH (70/30, *v/v*) and 1 mL of EtOAc/H_2_SO_4_ (100 mL/200 ng, *v/w*) at 50°C for 1 h. Subsequently, 0.5 mL of methanolic NaOH (1 M) were added, the mixture was evaporated as described above, and reconstituted with 5 mL of water. A third LLE with TBME was conducted to extract the formerly sulfo-conjugated steroids (fraction Sulf). This was followed by alkalization of the aqueous layer with 300 μL of 6 M KOH and an incubation step at 60°C for 15 min. Finally, alkaline-labile steroids comprising potential cysteine conjugates were extracted by the last LLE (fraction Cys).

The four resulting TBME extracts per sample were evaporated to dryness and the fractions of glucuronides and sulfates further purified by HPLC. For that purpose, samples were reconstituted in 100 μL ACN/H_2_O (50/50, *v*:*v*), and the entire volume was injected into an Agilent 1100 HPLC-UV system (Waldbronn, Germany) equipped with a X Bridge Shield RP18 column (4.6 × 250 mm) with 5 μm particle size (Waters, Eschborn, Germany). UV signals were acquired at 195 and 360 nm. Gradient elution was conducted as follows with a flow rate of 1 mL/min: Starting at 20% ACN/80% water, the gradient increased to 100% ACN within 25 min, was held for 10 min, and re-equilibrated for 5 min. With support of a Foxy R1 automatic fraction collector (Axel Semrau, Sprockhövel, Germany), the following HPLC sub-fractions were produced using the retention time markers shown in brackets. I: 3.00–10.00 min, II: 10.01–13.50 min (Tren, Epitren), III: 13.51–14.80 min (T), IV: 14.81–17.00 min (EpiT, DHEA, 5b, 5a, ETIO), V: 17.01–19.50 min (PD), VI: 19.51–24.50, and VII 24.51–33.00 min (16 EN).

The eluted HPLC sub-fractions were evaporated to dryness. Derivatization of all fractions derived from HPLC clean-up, as well as the fractions containing the cysteine adducts and free steroids was performed in accordance with the applied chromatographic system as described below.

#### Derivatization Techniques

##### Acetylation

For acetylation, samples were reconstituted in 75 μL of pyridine and 75 μL of acetic anhydride and derivatized for 1 h at 70°C. Subsequently, the derivatization mixture was evaporated. Samples were subjected to GC-TC-IRMS/MS (section GC-TC-IRMS/MS Setup), GC-EI-HRMS (QTOF) (section GC-EI-HRMS (QTOF) Setup) and LC-ESI-HRMS (section LC-ESI-HRMS (LC Orbitrap) Setup) analysis.

##### Trimethylsilylation

For trimethylsilylation, samples were reconstituted in 80 μL of MSTFA:NH_4_:ethanethiol (1000:2:3, *v*:*w*:*v*), incubated at 60°C for 45 min (Mareck et al., 2004, 2008), and measured as described in section GC-EI-HRMS (Orbitrap) Setup.

#### Instrument Methods

##### GC-TC-IRMS/MS setup

After evaporation of the derivatization mixture, the acetylated samples were reconstituted in an appropriate volume of cyclohexane (typically 20 μL) for GC-TC-IRMS/MS analysis. Analysis was performed on a Delta V Plus IRMS coupled via a GC Isolink CNH for thermal conversion at 1,450°C with a ceramic reduction reactor and ConFlow IV to a Trace 1310 GC (Thermo, Bremen, Germany). Chromatographic separation was accomplished on a DB-17 MS column (30 m × 0.25 mm) with a film thickness of 0.25 mm. The temperature gradient was as follows: The temperature remained constant at 100°C for 1.5 min and increased with 40°C/min to 240°C and subsequently with 5°C/min to 320°C with a hold time of 2 min. Samples were injected in splitless mode at 300°C with an injection volume of 5 μL. A single taper inlet liner (900 μL volume, 4 mm inner diameter, 6.47 mm outer diameter, 78.5 mm length) with glass wool from Agilent (part number: 5190-2293) was used. After passing the GC column, the flow was split by a ratio of approximately 1:10 to an ISQ single quadrupole mass spectrometer (Thermo, Bremen, Germany). Data acquisition and processing was accomplished using Isodat 3.0 and Xcalibur 2.2 software (Thermo, Bremen, Germany).

##### GC-EI-HRMS (QTOF) setup

Following GC-TC-IRMS/MS analysis, the acetylated samples were diluted to a final volume of 200 μL cyclohexane and subjected to GC-EI-HRMS measurements on an Agilent 7200 QTOF system hyphenated to an Agilent 7890A gas chromatograph (Santa Clara, CA). The chromatography setup was equivalent to GC-TC-IRMS/MS described above (section GC-TC-IRMS/MS Setup), including the same analytical column and the same temperature program. The injection volume was reduced to 4 μL. Data were acquired within a range of *m*/*z* 50–800 with an acquisition rate of 5 spectra/s and evaluated with MassHunter software (version B.06, Agilent). Mass calibration was performed before and during each analytical batch.

##### GC-EI-HRMS (Orbitrap) setup

The trimethylsilylated samples were injected (2 μL injection volume) into a Q Exactive GC Orbitrap (Thermo, Bremen, Germany). Due to the different derivatization technique, the system was operated with modified chromatographic conditions adapted from routine protocols (Thevis et al., 2011). The GC was equipped with a HP-Ultra 1 column (17 m × 0.2 mm) of 0.11 mm film thickness. The temperature program started at 180°C and raised with 3°C/min to 240°C and with 40°C/min to 320°C, where it remained constant for 2 min. Samples were injected in split mode with a split flow of 5 mL/min. The mass range for full MS experiments was *m*/*z* 50–700 and a resolution of 60,000 FWHM was applied. Data were evaluated with Xcalibur software.

##### LC-ESI-HRMS (LC Orbitrap) setup

For LC-ESI-HRMS measurements, the acetylated samples were evaporated and reconstituted with 100 μL of ACN/H_2_O (50:50, *v*/*v*) acidified with 0.1% FA. A Vanquish UHPLC (Thermo, Bremen, Germany) equipped with a Poroshell 120 EC-C8 (2.7 μm, 3 × 50 mm) (Agilent, Santa Clara, CA) was hyphenated to a Q Exactive HF-X (Thermo, Bremen, Germany). ACN and ultrapure water both containing 0.1% FA were employed as solvents, and the flow rate was set to 400 μL/min. A volume of 5 μL was injected per sample. The LC gradient run was as follows: Starting at 40% ACN, it was increased to 99% within 9 min, and hold for further 3 min until re-equilibration, yielding a total analysis time of 15 min. MS experiments comprised a full scan (*m*/*z* 200–800), AIF (all ions fragmentation), and PRM in positive ionization mode at a resolution of 60,000 FWHM. For PRM experiments, the isolation window was set to *m*/*z* 0.4 and in the higher-energy collisional dissociation (HCD) cell, collision energies of 20, 30, 35 or 40 eV were applied. For pseudo MS^3^ experiments, the source induced dissociation (SID) energy was set to 20 eV.

### Analysis of Conjugated Steroids

For the analysis of intact conjugated steroids, samples were diluted 1:1 with ultrapure water. In order to perform PRM experiments, selected samples were 5-fold pre-concentrated by SPE as described in section 2.3.1, and reconstituted in 50 μL 10% aqueous ACN. The setup for the LC-ESI-HRMS (Orbitrap) system was similar to the settings described in chapter LC-ESI-HRMS (LC Orbitrap) Setup, but employing a modified gradient. Starting at 1% ACN (containing 0.1% FA), the gradient increased to 40% ACN within 9 min, to 99% until 10.9 min, and to 1% until 11 min. The system was re-equilibrated for 3 min. Full MS, AIF, and PRM experiments were conducted and ESI with positive polarity was applied. In selected experiments, also negative ionization was used, which is explicitly indicated in the corresponding data sets.

### Synthesis of Trenbolone-diol

Trenbolone-diol was synthesized by reduction of Tren under argon atmosphere. To 10 mg of Tren dissolved in 10 mL of anhydrous THF, 100 μL of potassium tri-sec-butylborohydride (1 M in THF) were added under constant stirring. After 15 min, the reaction was stopped with 10 mL of ultrapure water, and subsequently, a LLE with 20 mL of TBME was performed. An aliquot of the yielded products was acetylated as described in section Acetylation.

## Results and Discussion

### Metabolite Identification With GC-TC-IRMS

The use of GC-TC-IRMS allows for comprehensive metabolite studies, particularly when investigating biotransformation products of deuterated compounds.

**Figure 3:**
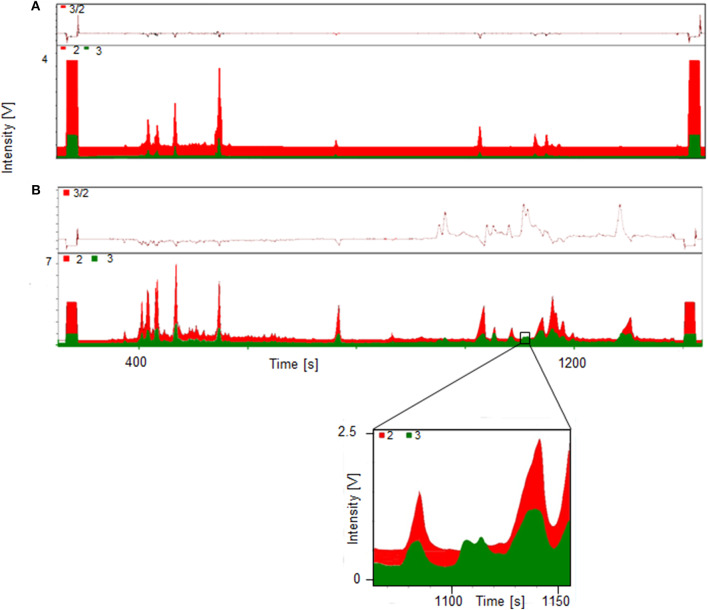
GC-TC-IRMS chromatograms of HPLC fraction II Gluc of a pre- **(A)** and 45 h post- **(B)** administration sample collected following ingestion of 10 mg of d_5_-TREN.

A significant increase of the ratio *m*/*z* 3 to *m*/*z* 2 indicates the presence metabolites of the administered substance, in this case trenbolone. Exemplary GC-TC-IRMS chromatograms of both a pre- and post-administration sample are displayed in Figure 3. The upper part (A) shows the chromatogram of the fraction containing the hydrolyzed glucuronides (fraction Gluc) of a pre-administration sample with deuterium levels at natural abundance. By contrast, the lower chromatogram (B) shows a sample collected 45 h following drug administration-, and several peaks of deuterated molecules corresponding to metabolites of Tren are visible. In the inset, details of the metabolites eluting at retention times between 1108 and 1110 s are shown.

### Trenbolone Metabolites Identified by LC-ESI-HRMS

Selected HPLC fractions exhibiting signals of deuterated compounds of considerable abundance were additionally analyzed by LC-ESI-HRMS(/MS). For metabolite identification, mass-to-charge-ratios were computed by *in silico* prediction and their presence or absence was assessed in the post-administration samples. Promising metabolites, their corresponding HPLC (sub-)fractions, retention times for LC-ESI-HRMS, exact masses, calculated elemental compositions, excretion forms, and detection windows are summarized in Table 1. Obtained elemental compositions were calculated within a maximum permitted mass error of ±4 ppm. Metabolites were determined in HPLC sub-fractions I, II, IV, and V. The number sequence corresponds to increasing retention times and decreasing polarity of the metabolites after hydrolysis, but before acetylation.

**Table 1:** Deuterated TREN metabolites and diagnostic product ions (CE = 20 and CE = 30 eV) after hydrolysis of phase-II metabolites, HPLC fractionation and acetylation identified by LC-ESI-HRMS.

**#**	**HPLC fraction**	**Retention time HFX [min]**	**Exact mass [*m*/*z*]**	**Elemental composition**	**Error [ppm]**	**Excretion form^a^**	**Detection window [h]**	**Diagnostic production [*m*/*z*]**	**Elemental composition**	**Error [ppm]**	**Diagnostic production [*m*/*z*]**	**Elemental composition**	**Error [ppm]**	**Diagnostic production [*m*/*z*]**	**Elemental composition**	**Error [ppm]**	**Diagnostic production [*m*/*z*]**	**Elemental composition**	**Error [ppm]**
1	l	4.39	301.2095	C_20_H_21_D_4_O${}_{2}^{+}$	−1.7	1_Sulf	45	259.2000	C_18_H_19_D_4_O^+^	2.1	241.1890	C_18_H_17_D${}_{4}^{+}$	0.5						
						1_Cys	45												
2	I	5.25	301.2091	C_20_H_21_D_4_O${}_{2}^{+}$	−3.0	2_Sulf	45	259.1999	C_18_H_19_D_4_O^+^	1.7	241.1890	C_18_H_17_D${}_{4}^{+}$	0.5						
						2_Cys	118												
3	II	3.33	318.2112	C_20_H_20_D_5_O${}_{3}^{+}$	−0.0	3_Gluc	21	276.2003	C_18_H_18_D_5_O${}_{2}^{+}$	−1.2	258.1896	C_18_H_16_D_5_O^+^	−1.8						
4	II	3.68	318.2112	C_20_H_20_D_5_O${}_{3}^{+}$		4_Gluc	45	276.2004	C_18_H_18_D_5_O${}_{2}^{+}$	−0.9	258.1897	C_18_H_16_D_5_O^+^	−1.5						
5	II	3.47	373.1944	C_22_H_21_D_4_O${}_{5}^{+}$	−1.0	5_Gluc	94	331.1837	C_20_H_19_D_4_O${}_{4}^{+}$	−1.8	313.1731	C_20_H_17_D_4_O${}_{3}^{+}$	−2.0	289.1731	C_18_H_17_D_4_O${}_{3}^{+}$	−1.8	271.1625	C_18_H_15_D_4_O${}_{2}^{+}$	−1.7
						5_Sulf	70												
6	II	3.56	313.1731	C_20_H_17_D_4_O${}_{3}^{+}$	−1.7	6_Gluc	94	271.1628	C_18_H_15_D_4_O${}_{2}^{+}$	−0.1	253.1523	C_18_H_13_D_4_O^+^	−0.8	227.1366	C_16_H_11_D_4_O^+^	−1.1			
						6_Sulf	70												
7	II	4.95	357.1995	C_22_H_21_D_4_O${}_{4}^{+}$	−1.0	7_Gluc	118	315.1888	C_20_H_19_D_4_O${}_{3}^{+}$	−1.5	297.1783	C_20_H_17_D_4_O${}_{2}^{+}$	−1.4	273.1784	C_18_H_17_D_4_O${}_{2}^{+}$	−1.2	255.1676	C_18_H_15_D_4_O^+^	−2.2
8	II	5.03	357.1996	C_22_H_21_D_4_O${}_{4}^{+}$	−0.7	8_Sulf	118	315.1888	C_20_H_19_D_4_O${}_{3}^{+}$	−1.5	297.1784	C_20_H_17_D_4_O${}_{2}^{+}$	−1.1	273.178	C_18_H_17_D_4_O${}_{2}^{+}$	−2.6	255.1678	C_18_H_15_D_4_O^+^	−1.4
						8_Cys	45												
9	IV	1.87	273.1787	C_18_H_17_D_4_O${}_{2}^{+}$	−0.1	9_Gluc	142												
						9_Sulf	94												
10	IV	3.35	313.1736	C_20_H_17_D_4_O${}_{3}^{+}$	−0.1	10_Sulf	94	271.1631	C_18_H_15_D_4_O${}_{2}^{+}$	0.1	253.1528	C_18_H_13_D_4_O^+^	1.2	227.1370	C_16_H_11_D_4_O^+^	0.7			
						10_Cys	45												
11	V	1.58	313.1736	C_20_H_17_D_4_O${}_{3}^{+}$	−0.1	11_Sulf	45	271.1635	C_18_H_15_D_4_O${}_{2}^{+}$	0.1	253.153	C_18_H_13_D_4_O^+^	2.0	227.1374	C_16_H_11_D_4_O^+^	2.4			
12	-	2.55	273.1788	C_18_H_17_D_4_O${}_{2}^{+}$	0.3	12_Cys	70	255.1685	C_18_H_15_D_4_O^+^	1.4	229.1531	C_16_H_13_D_4_O^+^	2.6						
13	-	4.83	359.2152	C_22_H_23_D_4_O${}_{4}^{+}$	−0.8	13_Cys	118	317.2054	C_20_H_21_D_4_O${}_{3}^{+}$	1.2	299.1946	C_20_H_19_D_4_O${}_{2}^{+}$	0.8	257.1840	C_18_H_17_D_4_O^+^	0.8	239.1737	C_18_H_15_D${}_{4}^{+}$	1.9

a *De-conjugation under alkaline conditions expecting potential cysteine conjugates*.

Altogether, a total of 20 phase-II metabolites of relevant traceability was identified. Almost all metabolites were found to be eliminated as differently conjugated products including the well-characterized glucuronic acid and sulfate conjugates, as well as alkaline-labile phase-II metabolites. De-conjugation under alkaline conditions is less established for human metabolism, but has already been assessed in preventive doping research to generate cysteine conjugates (Fabregat et al., 2010, 2013; Pozo et al., 2010; Gomez et al., 2014). Especially for Tren, cysteine conjugates are supposed to be of relevance (Sobolevsky and Rodchenkov, 2015).

Presumed phase-I reactions comprised hydroxylation, dehydrogenation, dehydration, and reduction of a keto to a hydroxyl moiety or *vice versa*. Most metabolites were identified as the 4-fold deuterated isomers of the metabolites although 5-fold deuterated Tren was administered. This suggests that a metabolic conversion occurs predominantly within the steroidal A or B ring. The 5-fold deuterated isomers of the well-known glucuronic acid-conjugated metabolites Tren (metabolite 4) and EpiTren (metabolite 3) were confirmed in the respective Gluc fraction and were unambiguously identified by retention time and product ion mass spectra in comparison to reference material.

**Figure 4:**
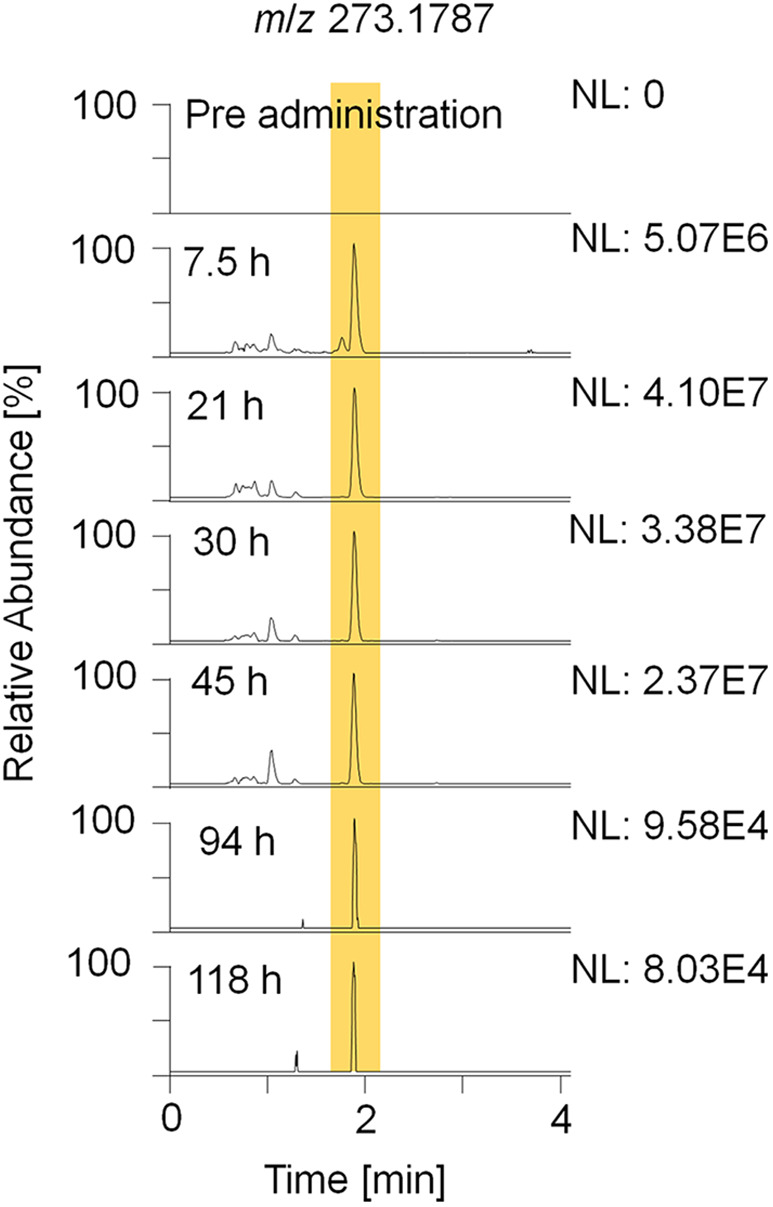
EIC of *m*/*z* 273.1787 (± 5 ppm) representing metabolite 9_Gluc after hydrolysis and acetylation analyzed by LC-HRMS in +ESI full MS mode.

For sports drug testing, especially long term metabolites with extended detection windows are of great interest. Within this study, metabolites 7, 8, 9, and 11 were found to have detection times between 118 h (5 days) and 142 h (6 days). Extracted ion chromatograms (EIC) displaying metabolite 9_Gluc in pre- and post-administration samples are depicted in Figure 4. The hydrolyzed metabolites 7 (excreted as glucuronide) and 8 (excreted as sulfate) are considered as isomers due to their identical elemental composition and their minor difference in retention time. Since metabolite 7/8 and metabolite 9 are excreted in two conjugation forms, in particular as glucuronides and sulfates, they were chosen for further characterization (see section Production Ion Mass Spectra).

### Metabolite Characterization

#### Product Ion Mass Spectra

Besides accurate mass measurements verifying the deuterium content, all identified metabolites were characterized by product ion mass spectra (MS^2^) obtained from PRM experiments. Analysis was performed in ESI positive mode, as it was found to produce more characteristic product ion mass spectra compared to those obtained following negative ionization (Rzeppa et al., 2015). Collision induced dissociation (CID) was accomplished at different collision energies ranging from 20 to 40 eV.

**Figure 5:**
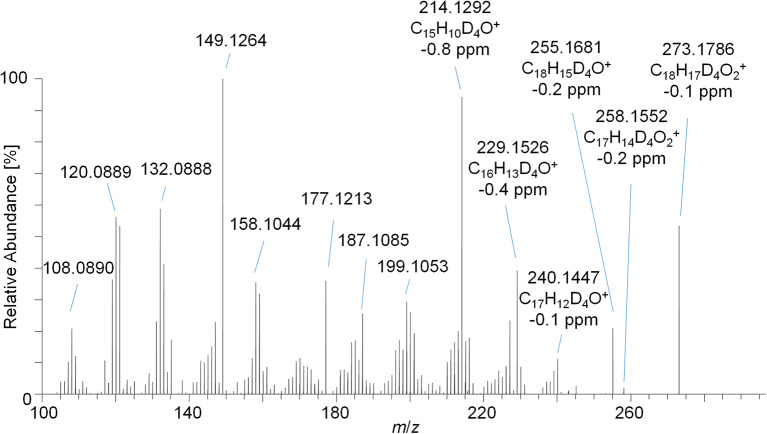
PRM mass spectrum of *m*/*z* 273.1787 at 35 eV representing metabolite 9_Gluc after hydrolysis and acetylation analyzed by LC-HRMS.

The PRM mass spectrum of metabolite 9_G after hydrolysis and acetylation is illustrated in Figure 5 as an example, the diagnostic product ions, elemental compositions, and mass errors of the other metabolites are listed in Table 1. From the elemental composition and the MS/MS-experiments, metabolite structures were postulated. Metabolite 5 is tentatively assigned to a hydroxyl-metabolite of Tren, which was substantiated by the 2-fold loss of an acetyl moiety [neutral loss of *m*/*z* 42 (Ac) and *m*/*z* 60 (AcOH)]. For this metabolite, the generation of a fourth double bond by oxidation of a tertiary carbon atom is additionally required.

The metabolites 7 and 8 are tentatively assigned to derivatives of Tren that result from the reduction of the 3-oxo functionality of the anabolic steroid as supported by the characteristic and repeatedly occurring losses of acetyl moieties. Further, the presence of two additional double bonds (of unknown position) is postulated.

The elemental composition of metabolite 9 indicates a potential metabolic conversion to trenbolone-diketone. The metabolite was not derivatized by pyridine/acetic anhydride, which corroborates the absence of hydroxyl functions. In accordance with the current state of knowledge, free hydroxyl groups are acetylated under the applied conditions, but -oxo functions are not affected; however, failed derivatization due to steric hindrance cannot be excluded (Piper et al., 2008).

The detection of trenbolone-diketone, also known as 17-keto-trenbolone or trendione, was already reported in 1991 (Spranger and Metzler, 1991) for *in vivo* samples, and the compound could be generated *in vitro* by the same group by using human liver microsomes (Metzler and Pfeiffer, 2001).

Noteworthy, an additional abundant signal of *m/z* 97.0652 corresponding to C_6_H_9_O as elemental composition (±4.2 ppm) was observed in the PRM spectra. Since trenbolone-diketone is deuterated in the A- and B-ring, the signal has to be derived from the steroidal C- or D-ring. Structural elucidation of the equivalent diagnostic ion has been accomplished for androst-4-en-3-one-based steroids, but this structure cannot be immediately transferred to the herein investigated molecule as it was shown to originate from the steroidal A-ring (Thevis et al., 2012).

#### Synthesis of Trenbolone-diol

**Figure 6:**
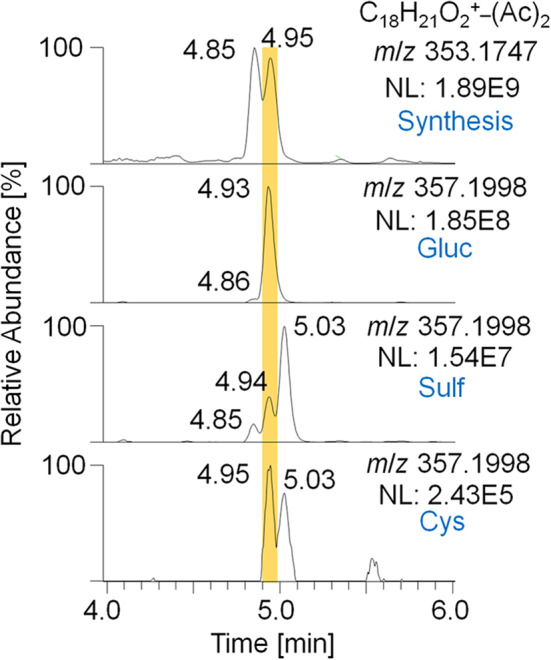
EIC of *m*/*z* 353.1747 (± 5 ppm) representing the 2-fold dehydrogenated trenbolone-diol derivatives obtained by in-house synthesis and *m*/*z* 357.1998 (± 5 ppm) representing the deuterated metabolites 7/8 eliminated as glucuronide, sulfate, and cysteine conjugate after hydrolysis and acetylation analyzed by LC-HRMS in +ESI full MS mode.

For the metabolites 7_Gluc and 8_Sulf, which were proposed to represent trenbolone-diol derivatives, further in-depth studies were conducted. In order to obtain a reference mass spectrum of trenbolone-diol, an in-house synthesis was accomplished by reducing Tren with potassium tri-sec butylborohydride. Two isomers of trenbolone-diol were successfully synthesized and PRM spectra of the free and acetylated forms were acquired. Remarkably, also 1-fold and 2-fold dehydrogenated trenbolone-diol derivatives were obtained as byproducts in two isomeric forms despite the employed reducing conditions. The 2-fold dehydrogenated trenbolone-diol derivative was found to be analog to the predicted metabolite 7/8 after hydrolysis. In Figure 6, the EIC of the synthesized 2-fold dehydrogenated trenbolone-diol derivative (A) is compared to the metabolites identified in the post-administration urine samples (B). The retention times (4.85 and 4.95 min) of the synthesized products are in accordance with the postulated metabolites. Both synthesized isomers were present in the glucuronide and the sulfate fraction. While the metabolite at 4.95 min is the most prominent isomer eliminated as glucuronide, the sulfated analog with the longest detection window at 5.03 min was not synthesized. The potential cysteine conjugates are less valuable due to their short detection window.

**Figure 7:**
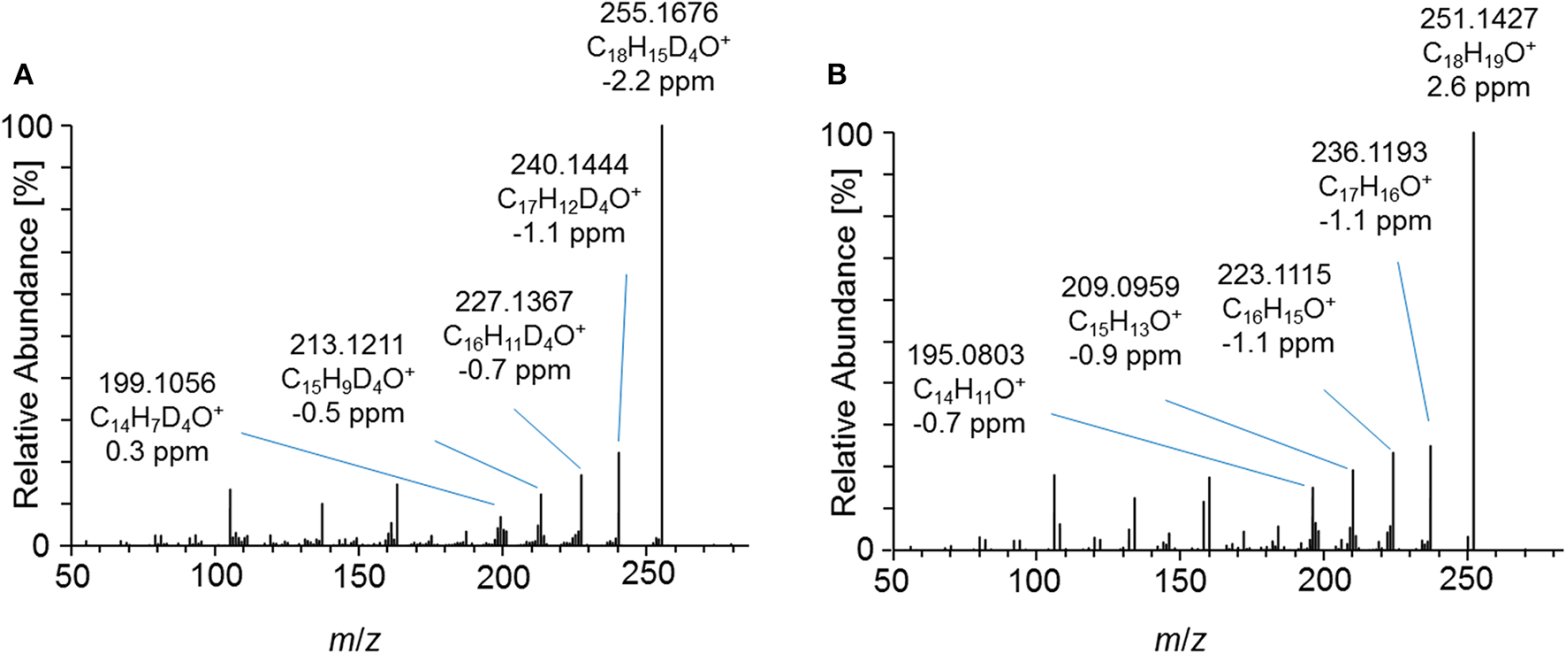
PRM mass spectrum of **(A)** *m*/*z* 357.1995 at 40 eV representing the deuterated metabolite 7_Gluc after hydrolysis and acetylation and **(B)** *m*/*z* 353.1747 at 40 eV representing the synthesized 2-fold dehydrogenated trenbolone-diol derivatives at 4.95 min by LC-ESI-HRMS.

Product ion mass spectra of the acetylated 2-fold dehydrogenated trenbolone-diol derivative isomer at 4.95 min and the acetylated metabolite 7_Gluc after hydrolysis are in good agreement as demonstrated in Figure 7. The observed mass shift of four Da is caused by the 4-fold deuteration of the metabolite. The PRM mass spectra of the three hydrolyzed metabolites were highly comparable in all excretion forms. As a consequence, they are assumed to be different isomeric forms of the 2-fold dehydrogenated trenbolone-diol derivative. Isomers can be attributed to both stereo centers at C3 and C17, as well as various positions of the additional double bonds.

#### Phase-II Conjugates

The sample preparation comprising SPE, LLE, hydrolysis, HPLC fractionation, and derivatization is very elaborate and may be accompanied by analyte losses and/or the formation of artifacts during derivatization (Piper et al., 2019). Moreover, the herein employed approach of analyzing acetylated steroids by LC-ESI-HRMS is certainly unconventional.

In sports drug testing, the implementation of new analytes/metabolites into existing methods is of great importance to enable a specific and sensitive detection of the target molecules as well as adequate detection windows. Currently, three different methods are routinely used for the analysis of doping control samples, where an implementation of novel Tren metabolites appears feasible: (a) measurement of analytes hydrolyzed and derivatized as TMS derivatives with GC-EI-MS/MS, (b) measurement of hydrolyzed, but not acetylated analytes, (c) measurement of intact phase-II metabolites with LC-ESI-HRMS. Since the ionization efficacy of steroid diols with low proton affinity such as metabolites 7/8 by ESI is usually limited, the existing LC-ESI-HRMS approach was not considered as the preferred option. When using LC-ESI-HRMS, it is generally advisable to measure the intact phase-II conjugates, which also results in a reduced workload (Gomez et al., 2014).

**Figure 8:**
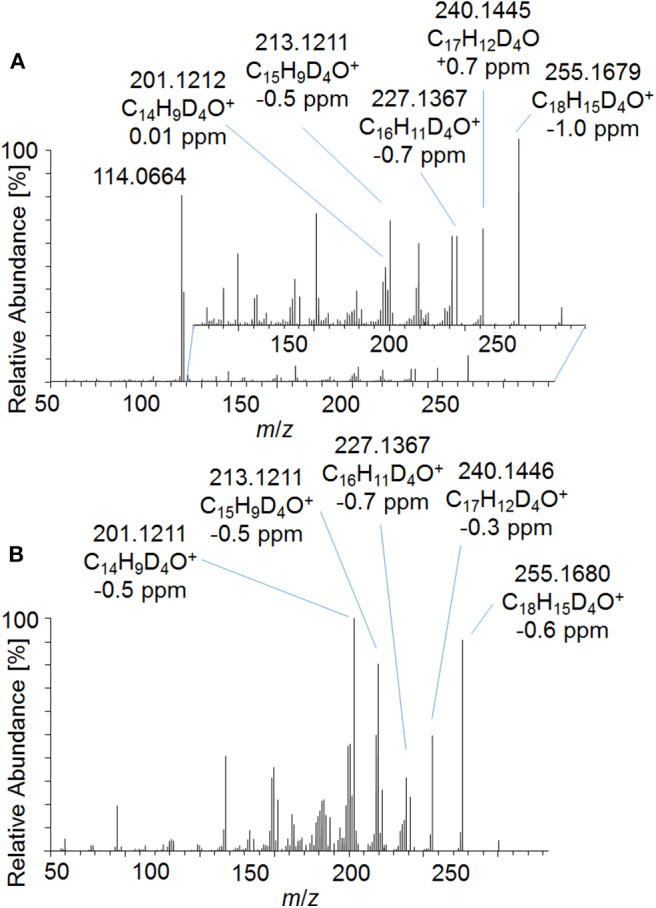
Pseudo MS^3^ mass spectra of **(A)** *m*/*z* 449.2108→273.1787 representing the glucuronidated metabolite 7_Gluc and **(B)** *m*/*z* 357.1995→273.1787 representing the metabolite 7_Gluc after hydrolysis and acetylation at SID = 20 eV and CID = 30 eV.

In order to investigate the new metabolites regarding their applicability to doping control routine testing, the intact phase-II metabolites were analyzed by LC-ESI-HRMS. For the potential Tren-diol derivatives (metabolite 7_Gluc), a peak corresponding to the singly glucuronidated metabolite was identified. The pseudo MS^3^ mass spectra of the intact glucuronide and the acetylated and hydrolyzed metabolite are compared in Figure 8. Both product ion mass spectra derived from *m*/*z* 273.1787 are in good agreement. Observed variations in the relative intensities are potentially caused by differences in the energetic status of both molecules resulting from the in-source dissociation process.

#### Comparison to Reference Material

**Figure 9:**
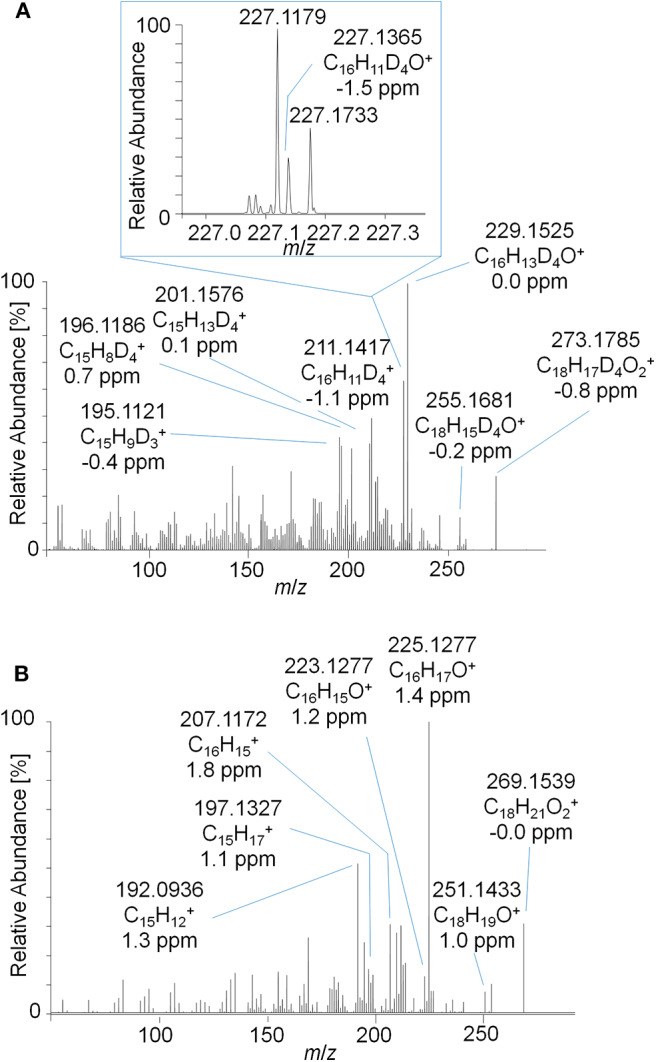
**(A)** Pseudo MS^3^ mass spectrum of *m*/*z* 353.1355→273.1787 at SID = 20 eV and CID = 30 eV representing the sulfated metabolite 9_Sulf and **(B)** PRM mass spectrum of *m*/*z* 269.1536 at 30 eV representing trenbolone-diketone reference material.

As outlined in above, the characterization of metabolite 9 yielded a potential diketone derivative of trenbolone. A commercially available reference standard of trenbolone-diketone, 4,9,11-estratriene-3,17-dione, was analyzed by LC-ESI-HRMS. The pseudo MS^3^ spectrum of the sulfated diketone metabolite in urine and the product ion mass spectrum of the reference standard are displayed in Figure 9. Again, the mass shift of four Da is caused by the 4-fold deuteration of the metabolite. The good agreement of both product ion mass spectra supports the assignment of metabolite 9_Sulf to a potential diketone derivative. It is noticeable that phase-II conjugation appears to occur via keto-/enol-tautomerism as described earlier also for androstenedione (Tajic and Kovacic, 1968; Goodall and James, 1979, 1981).

### Determination of Detection Windows for Published Long-Term Metabolites

**Figure 10:**
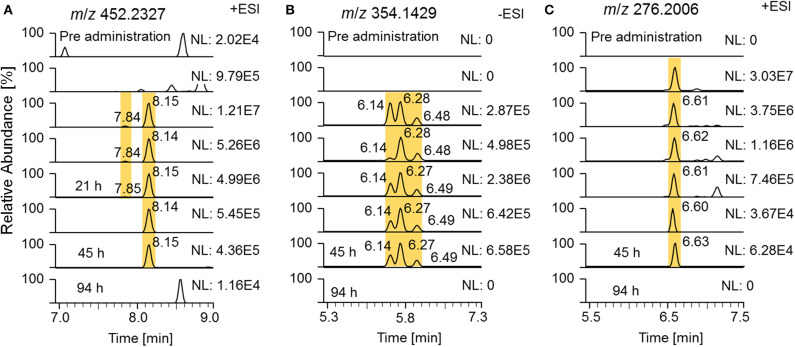
EIC of **(A)** *m*/*z* 452.2327 (± 5 ppm) representing deuterated trenbolone glucuronide, **(B)** *m*/*z* 354.1429 representing deuterated trenbolone sulfate, and **(C)** *m*/*z* 276.2006 representing deuterated trenbolone cysteine adduct by LC-HRMS.

State-of-the-art routine doping control methods are primarily based on epitrenbolone, trenbolone glucuronide and epitrenbolone glucuronide (De Boer et al., 1991; Schänzer, 1996; Brun et al., 2011). In addition, trenbolone sulfate and the trenbolone cysteine adduct have been published (Rzeppa et al., 2015; Sobolevsky and Rodchenkov, 2015). While no detection windows for the sulfate conjugate have been described yet, limited data sets are available for the glucuronide conjugate. An early Tren metabolism study used radioactive labeling and investigated the urinary excretion. Spranger and Metzler detected 54% of the radioactivity within 26 h and 63% within 72 h post Tren administration, indicating a fast elimination (Spranger and Metzler, 1991). A publication from the former Russian anti-doping laboratory published the traceability of trenbolone administrations of 32 days when employing epitrenbolone glucuronide as target analyte; similarly the trenbolone cysteine adduct was detected for 32 days. The deuterated analogs of the described metabolites were also detected in this elimination study as illustrated in Figure 10 by means of respective EIC.

Epitrenbolone was detectable for 45 h and Tren for 21 h after oral administration of 10 mg of trenbolone. For the sulfate conjugate, three potential isomers were identified, two of which are obviously induced by epimerization at position 17. The third one remained unclear but might be attributed to a conjugate directed to position 3. This can be derived from the occurrence of the above described assumed conjugation of the diketone derivatives metabolite 9_Gluc and 9_Sulf, which appear to be conjugated via a steroidal keto group. All three isomers were detectable for 45 h following administration. The Tren cysteine conjugate was observed for 45 h on *m*/*z* 276.2006 referring to the steroid structure which is generated by in source dissociation. The intact phase-II metabolite at *m*/*z* 397.2204 was also visible, but for Figure 10, the mass-to-charge ratio of the variant with the longest detectability was chosen.

The results generated in this study corroborate the primary data of Spranger and Metzler, where Tren was identified as substance with a fast elimination. Inter-individual variations in the metabolism of the volunteers of the different studies are a conceivable explanation for deviating results, but investigation of a larger population appear necessary and warranted to further substantiate the observations.

### Limitations of Combining Data Obtained From GC-TC-IRMS/MS, LC-ESI-HRMS, and GC-EI-HRMS

In previous publications, GC-TC-IRMS/MS has been successfully employed in support of steroidal metabolite identification, especially when combined with GC-EI-HRMS (Thevis et al., 2013; Piper et al., 2016a,b, 2018, 2019).

**Figure 11:**
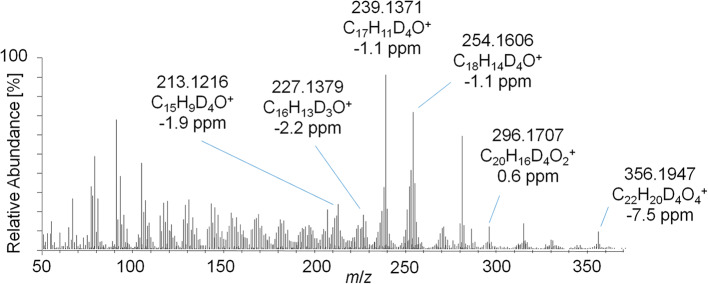
GC-EI-HRMS full MS mass spectra of trenbolone-diol metabolites excreted as glucuronides (7_Gluc).

This concept was adapted to this study, and referring to Figure 3, two metabolites were identified by using the conventional approach. The first peak at 1108 s was identified as the glucuronic acid conjugate of the well-characterized metabolite EpiTren by comparing the retention time and mass spectrum to reference material. The metabolite at 1110 s was formerly unknown and further characterized by GC-EI-HRMS (TOF) analysis. The resulting mass spectrum is shown in Figure 11. Under consideration of the different ionization and dissociation mechanisms, this mass spectrum was found to correspond to that of the deuterated 2-fold dehydrogenated trenbolone-diole derivative characterized by LC-ESI-HRMS (Figure 7).

Unfortunately, the described approach could not be applied to all metabolites. The quality of analytical data generated by the GC-TOF system suffered from an intense fragmentation, which led to a reduced abundance or absence of the molecular ion, complicating structural elucidation of new metabolites (Thevis et al., 2013; Piper et al., 2016a,b, 2018, 2019).

**Figure 12:**
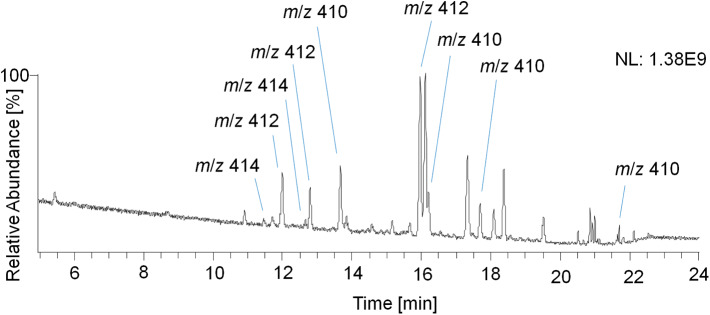
GC-EI-HRMS full MS chromatogram of epitrenbolone reference material as TMS-derivatives.

Therefore, a GC Orbitrap system with an alternative ion source design favoring higher abundances of ions with larger *m/z* values was used, and the measured TMS derivatives further exhibited a reduced in-source fragmentation compared to acetates (Piper et al., 2018, 2019). In addition, trimethylsilylation of steroidal analytes is routinely used, thus offering a platform for implementing potential new target analytes into sports drug testing methods. However, the analysis of TMS derivatives with the GC Orbitrap remained challenging as the analysis of reference material of trenbolone, epitrenbolone and d_5_-trenbolone introduced as TMS-derivatives resulted in a variety of signals presumably caused by derivatization and thermal degradation artifacts. Consequently, no unreported metabolites could be identified. For the TMS derivatives of trenbolone and epitrenbolone, a signal at *m*/*z* 414.2405 representing the molecular ion is expected. Moreover, peaks at *m*/*z* 412.2248 and *m*/*z* 410.2092 have been reported, which belong to products eliminating two or four hydrogens (De Boer et al., 1991; Ayotte et al., 1996). As shown in Figure 12, several additional peaks of unknown origin were observed in the reference material, which suggested a significantly limited utility of this setup for identifying additional metabolites in urine.

It has to be taken into account that the herein investigated molecule possesses poor gas chromatographic properties, and the highly conjugated 4,9,11-triene-3-one structure results in derivatization artifacts with low thermal stability. Other structurally related molecules such as the designer steroid tetrahydrogestrione are not detectable in urine as TMS-derivatives as well, and remained hidden until its first identification as performance-enhancing drug in 2003 by means of LC-MS/MS (Catlin et al., 2004; Thevis et al., 2005a). In other words, Tren has a documented history of challenging analytics as corroborated by a variety of assessed derivatization techniques (De Boer et al., 1991; Ayotte et al., 1996; Casademont et al., 1996; Marques et al., 2007; Brun et al., 2011; Parker et al., 2012).

LC-ESI-MS was found to be suitable for the quantification of Tren (Thevis et al., 2005a,b, 2009) in the past, and common routine doping control procedures nowadays utilize LC-ESI-MS/MS for analytes of this and related structure. As a consequence, also here a LC-ESI-HRMS system was used to overcome the limitations regarding the mass resolution and the presence of the molecular ion. Furthermore, a derivatization by acetylation improved the proton affinity for ESI positive ionization mode, and especially the ionization of the trenbolone-diol derivatives metabolite was found to be strongly increased.

The advantage of the untargeted GC-TC-IRMS approach is unfortunately not as pronounced as in previous studies as aligning LC and GC chromatograms and (presumed) molecular ions for target analyte characterization is difficult.

## Conclusion

In order to enhance the retrospectivity and sensitivity of analytical approaches targeting trenbolone misuse in sport, a comprehensive *in vivo* metabolism study was performed. An approach utilizing stable isotope-labeled substrates facilitating the investigation of biotransformations by GC-TC-IRMS was employed. While the strategy proved straightforward in earlier studies, trenbolone and its metabolic products presented comparably challenging target analytes due to their limited compatibility with gas chromatography. Nevertheless, by employing miscellaneous techniques of derivatization and chromatography, a total of 20 metabolites excreted as glucuronides, sulfates and potential cysteine conjugates were identified. Four metabolites, tentatively attributed to trenbolone-diketone and a 2-fold dehydrogenation product of trenbolone-diol, eliminated both as glucuronide and sulfate, were found to complement the existing urinary trenbolone metabolic pattern, offering detection windows of 6, respectively 5 days. Further characterization of these metabolites was conducted by pseudo-MS^3^ experiments and comparison to commercially available or in-house synthesized reference material. To verify or falsify the true added value of the herein identified trenbolone metabolites for routine doping controls, those samples that return suspicious or even adverse analytical findings for trenbolone using established approaches will be further investigated regarding the new potential target analytes. If a positive contribution will be observed, future studies to confirm tentatively assigned structures e.g., by nuclear magnetic resonance analysis after upscaling of the synthesis and an administration study of unlabeled trenbolone could be warranted. Moreover, it might be of interest to administer other doses of trenbolone and to investigate a larger population for examination of inter-individual variations.

## Data Availability Statement

The datasets generated for this study are available on request to the corresponding author.

## Ethics Statement

The administration study involving a human participant was approved by the Ethics Committee of the National Institute of Sports of Romania (Bucharest, Romania, #2283, 2016) according to the declaration of Helsinki. Informed consent was obtained from the volunteer.

## Author Contributions

MT and TP conceived conception and study design, contributed to data interpretation and discussion, and revised and edited the manuscript. MP conducted the sample preparation, measurements, data evaluation, and wrote the draft of the manuscript.

## Conflict of Interest

The authors declare that the research was conducted in the absence of any commercial or financial relationships that could be construed as a potential conflict of interest.
